# Injections of Glycerine and Carbolic Acid for the Preservation of Bodies for Dissection

**Published:** 1884-07

**Authors:** O. T. Freer

**Affiliations:** Munich


### ARTICLE V.

### INJECTIONS OF GLYCERINE AND CARBOLIC ACID FOR THE PRESERVATION OF BODIES FOR DISSECTION.

O. T. FREER, *M. D.*, *Munich.*

During a course of dissection in the anatomical department of the University of Munich, I observed that the material showed a remarkable resistance to decay, being still fresh at a period when the average subject of Chicago's dissecting rooms is too much decomposed for use. The parts which I was dissecting were in almost as perfect a condition after exposure to the air of a warm room for from two to four weeks, as when they were first given me; and those portions from which the skin had not been removed remained entirely unchanged.

On inquiry I learned from Professor Rudinger, Munich's anatomist, a man who has written numerous valuable works and done much for science by his original researches, that he preserved his subjects by injecting into the femoral artery a mixture of glycerine, carbolic acid, and alcohol, a method invented by himself and used here since 1872.

Since its introduction he had seen no bad effects from dissection wounds here.

He said that, according to the amount of injection-material used, subjects would keep from two to six months in a summer-temperature, the only necessity being moisture to prevent mummification.

The solutions used by Professor Rudinger are:

(*a*) For preserving bodies for long periods, three to six months: Glycerine, 40 parts; carbolic acid, crystalized, 11; alcohol, 8.

(*b*) For preserving bodies from two to three months: Glycerine, 80 parts; carbolic acid, 17; alcohol, 13.

The amount injected varies according to the length of time it is desired to preserve the subject, from two to four liters being ordinarily used; but an average body will readily contain six and more.

The advantages of this method are, that the tissues preserve their natural appearance and pliancy from the commencement of an ordinary dissection to the end. Students, therefore, need not hurry their dissection, even in warm weather, as decomposition is so slow that the parts dissected last are in as perfect a state as those seen first. The solutions mentioned above do not whiten the tissues, which retain their natural appearance to the end.

